# Delayed Appearance of Neuropathy After Inferior Shoulder Dislocation

**DOI:** 10.7759/cureus.72204

**Published:** 2024-10-23

**Authors:** Leslie A Jewett, Brian D Milman

**Affiliations:** 1 Emergency Medicine, CHI St. Vincent Hospital, Hot Springs, USA; 2 Emergency Medicine, University of Texas Southwestern Medical Center, Dallas, USA

**Keywords:** a case report, axillary neuropathy, delayed neuropathy, inferior shoulder dislocation, traumatic

## Abstract

Complications regarding inferior shoulder dislocations (ISD) are predominately rotator cuff injuries, neuropathies, and vascular insults. To our knowledge, there are no studies regarding the delayed appearance of neuropathies with inferior shoulder dislocations.

A 32-year-old previously healthy male presented with an inferior shoulder dislocation that required open reduction and internal fixation after failed attempts at closed reductions in the emergency room and operating room. At the time of discharge, the patient had no neurologic complaints, which continued to the two-week follow-up appointment. The patient did not complain of numbness or tingling until the six-week follow-up visit, at which point electromyography revealed a severe axillary nerve neuropathy.

Neuropathic complications following inferior shoulder dislocations to date have been detailed and described immediately after injury, with some resolving after reduction. The generally accepted theory regarding the pathophysiology of neuronal injury after trauma, Wallerian degeneration, does not account for the six-week delay in neuropathy demonstrated in this case report. This illustrates the importance of specifying the onset of neuropathies after inferior shoulder dislocation, which may lead to a more comprehensive theory regarding the pathophysiology of delayed presentations of neuropathy after inferior shoulder dislocation.

## Introduction

Shoulder dislocations are the most common joint dislocation among adults and are frequently seen in the emergency department [1]. The majority of these dislocations are classified as anterior. Inferior and posterior dislocations are less common, with incidences of 0.5% and 2% to 5%, respectively [1,2]. Classically, patients with inferior shoulder dislocations present with an abducted arm and an inability to adduct without significant pain [1]. However, an inferior shoulder dislocation can mimic a subcategory of glenohumeral dislocation known as subglenoid anterior dislocation. Differentiation is determined by the relation between the humeral shaft and the spine of the scapula [3]. In inferior dislocations, the humerus is parallel to the scapular spine. 

Complications following shoulder dislocation include fractures (bony Bankart lesions, Hill-Sachs lesions, and greater tuberosity fractures), rotator cuff tears, brachial plexopathy, and vascular compromise [4]. Given the low incidence of documented inferior shoulder dislocation, discussion of typically associated injuries and sequela must be taken with caution. In a 2018 literature review article, authors found only 199 cases. The largest case series since that time evaluated 38 patients and their sequela [4,5]. Neurologic injury was noted in 29% of patients in the 2018 review and 21% in the 2022 review [4,6]. The timing of the neurologic complaints was not specified in either review, though comments regarding the resolution of symptoms at the time of follow-up were noted. To our knowledge, this is the first published case specifying the delayed occurrence of neurologic symptoms after an inferior shoulder dislocation.

## Case presentation

A 32-year-old previously healthy male presented with acute right shoulder pain after a fall while running earlier in the evening. The patient reported intact sensation in the distal extremity, gross motor weakness, and limited range of motion at the acromioclavicular joint secondary to pain. He held his arm adducted at the shoulder with a flexed elbow and resisted any attempts to move it.

A physical exam was significant for an obvious deformity to the right upper extremity, palpable peripheral pulses, and capillary refill in less than two seconds. The patient's sensation was intact on the shoulder, lateral and medial forearm, and digits. 

**Figure 1:**
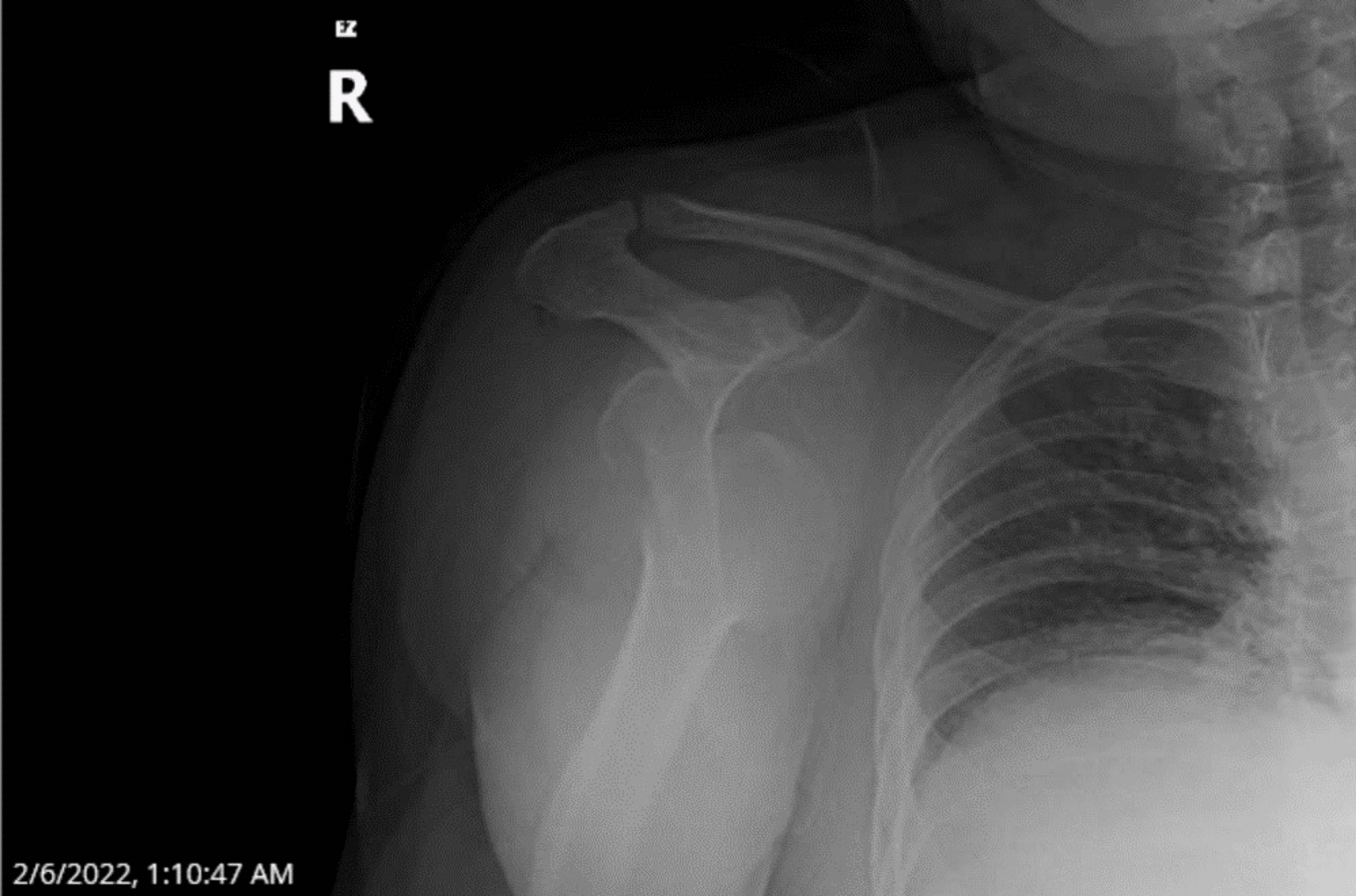
Assumed subglenoid anterior dislocation

X-ray images were concerning for subglenoid anterior dislocation, and the patient consented to a reduction in the ER (Figure 1).

**Figure 2:**
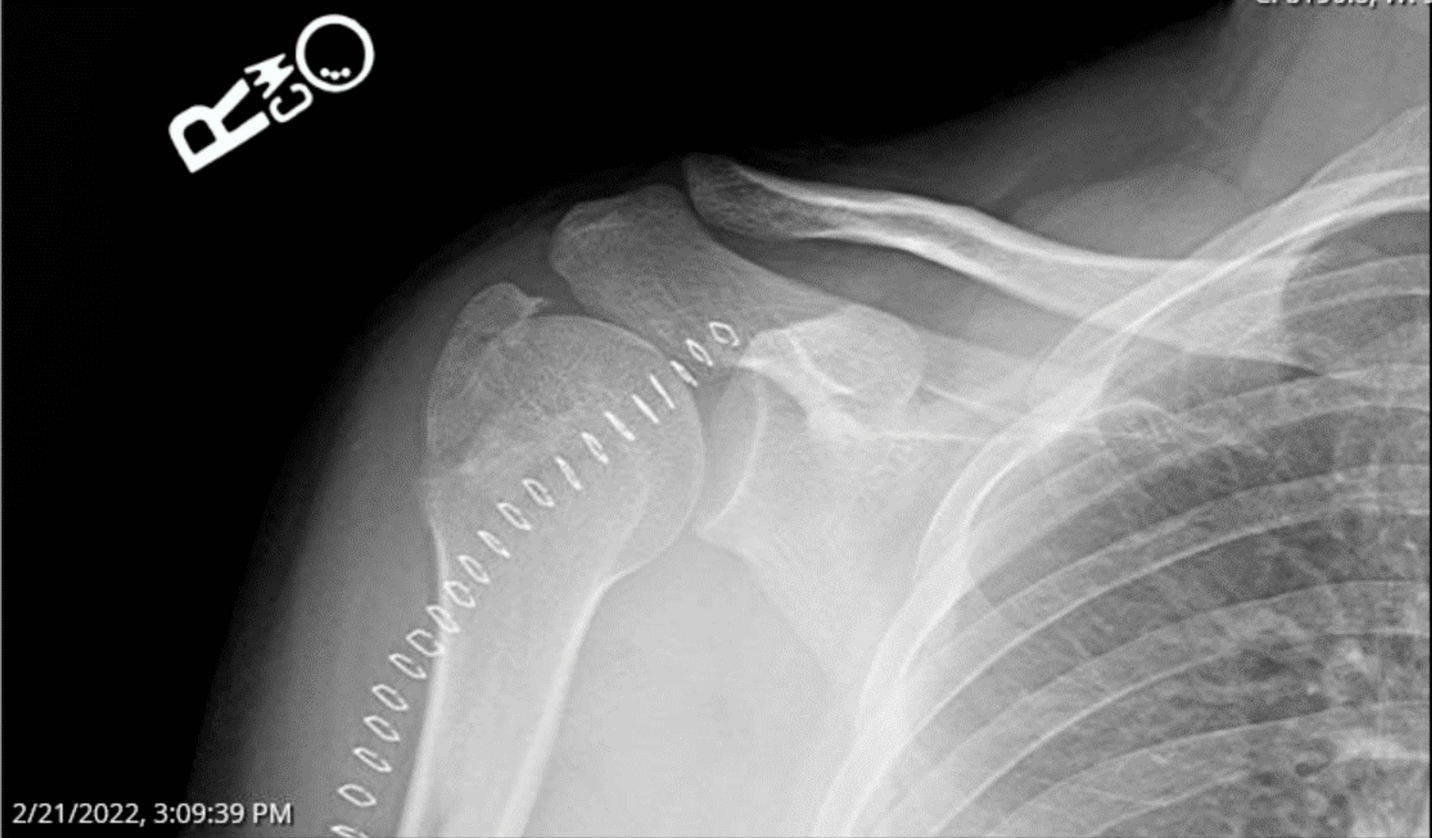
X-ray showing greater tuberosity fracture following open reduction and internal fixation

Attempts with traction-countertraction and the external rotation technique were unsuccessful in the ER. Closed reduction in the OR was unsuccessful despite the use of paralytics. Attempts at manual disimpaction of the fracture were unsuccessful, and the orthopedic surgeon used a threaded Steinmann pin placed in the humeral head, allowing her to push the humeral head inferiorly and posteriorly around the glenoid, which then allowed for successful reduction. Difficulty with closed reduction occurred because the fractured portion was locked on the inferior rim of the glenoid and unable to be moved. No damage to the rotator cuff muscles was noted intraoperatively. The postoperative diagnosis was a locked inferior shoulder dislocation as well as a greater tuberosity fracture (Figure 2).

The patient was discharged from the post-anesthesia care unit later that day with a follow-up visit two weeks later. Medications prescribed included opioids and non-steroidal anti-inflammatory drugs (NSAIDs). At the time of discharge and initial follow-up visit, he had no numbness or tingling in that extremity.

At the six-week follow-up clinic visit, the patient complained of numbness in the shoulder. Watchful waiting and physical therapy were decided on as the next steps. Electromyography was completed at the four-month mark and showed severe right axillary neuropathy with marked denervation and reduced recruitment in the deltoid with no evidence of carpal tunnel syndrome or ulnar neuropathy. Patient was informed regarding potential time to resolution vs. permanent and instructed to follow up as needed.

## Discussion

Discussions regarding pathophysiologic complications following inferior shoulder dislocation are limited as the incidence is only 0.5% of all dislocations [1,4]. This classic incidence was called into question by Wolf et al. by considering the pathway to dislocation and not solely the final position of the humeral head [7]. This case report supports that claim, as an inferior shoulder dislocation would not have been diagnosed if a closed reduction in the emergency department or the operating room had been successful. More advanced imaging after failed reductions may be obtained, which may assist with possible operative planning and a more detailed description of the injury. 

Neurologic sequela is relatively common in most shoulder dislocations, typically with symptoms resolving following reduction [7]. Following inferior shoulder dislocation, neuropathies occur in 21-29% of cases [4,6]. Specific neuropathies noted were the ulnar, radialis, and axillary. Axillary neuropathy is the most common neuropathy following inferior dislocation [6]. Susceptibility of the axillary nerve is potentially secondary to traction on the nerve while coursing through the quadrangular space [8]. Following reduction, there is a decrease in the stretch experienced by the nerve, which is consistent with the resolution of most neuropathies after reduction [8]. In some cases, when there is greater tension on the nerve, neuropathy may persist even after reduction. 

Here, we described a case of delayed neuropathy. The tension in the quadrangular space is not a plausible mechanism to explain this patient’s delayed presentation. Peripheral neuropathy secondary to trauma is considered Wallerian degeneration, where the axon experiences some type of compression, and the distal sections are left without nutrients from the cell body [9]. Postulating that the axons distal to the quadrangular space experienced enough swelling in the postoperative period is largely speculative, though there is a possibility of a missed axillary nerve neuropraxia at presentation or after reduction.

Limitations of this case report include no baseline electromyography to determine a definitive time of onset of neuropathy. Additionally, there is the possibility that the patient experienced another occult injury that led to the neuropathy. Further studies specifying the onset of neuropathies after inferior shoulder dislocation are warranted to enable a more comprehensive theory regarding the pathophysiology of delayed presentations of neuropathy after inferior shoulder dislocation.

## Conclusions

Neuropathic complications following inferior shoulder dislocations often occur immediately following injury and resolve after reduction. The generally accepted theory regarding the pathophysiology of neuronal injury after trauma, Wallerian degeneration, does not account for a six-week delay in the neuropathy demonstrated in this case report. This illustrates the importance of specifying the onset of neuropathies after inferior shoulder dislocation, which may lead to a more comprehensive theory regarding the pathophysiology of delayed presentations of neuropathy after inferior shoulder dislocation.
